# Strategies for RNA m^6^A modification application in crop improvement

**DOI:** 10.3389/fpls.2024.1477240

**Published:** 2024-10-15

**Authors:** Jun Tang, Xuemin Wang

**Affiliations:** Institute of Animal Sciences, Chinese Academy of Agricultural Sciences, Beijing, China

**Keywords:** RNA modification, gene expression, m^6^A modification, m^6^A application, crop improvement, m^6^A editing

## Introduction

The improvement of crop yield and quality is an eternal theme to face climate change and population growth. The key to improving crop varieties lies in precisely manipulating gene expression. Recent advancements in CRISPR/Cas9 technology have made gene knockout increasingly straightforward, yet for genes related to important agronomic traits, it is crucial to regulate their expression levels appropriately. Complete knockout often results in defects in other aspects. In addition, many agronomic traits require upregulation of target gene expression for their improvement. Therefore, the development of novel methods for precise upregulation or downregulation of gene expression, without altering gene protein sequences or introducing new genome fragments, will significantly bolster the technical foundation for crop genetic improvement.

*N* ^6^-methyladenosine (m^6^A) is the most abundant and reversible internal chemical modification in eukaryotic mRNA, which is installed, removed, and recognized by methyltransferases (writers), demethylases (erasers), and m^6^A-binding proteins (readers), respectively (Tang et al., 2023). Currently, two types of m^6^A methyltransferases have been identified in plants: multiprotein complexes and a single protein. The multiprotein complex includes MTA, MTB, FIP37, VIRILIZER (VIR), HAKAI, and HIZ2 (HAKAI interacting zinc finger protein 2), which catalyze the majority of m^6^A modifications in mRNA (Parker et al., 2021; Ruzicka et al., 2017; Shen et al., 2016; Zhang et al., 2022; Zhong et al., 2008). The single protein FIONA1 also exhibits methyltransferase activity in Arabidopsis (Wang et al., 2022; Xu et al., 2022), catalyzing approximately 10% of m^6^A modifications in mRNA. Several m^6^A demethylases, which belong to the Fe (II)/α - kg dependent dioxygenase superfamily, have been identified in plants, including Arabidopsis AtALKBH10B and AtALKBH9B (Martinez-Perez et al., 2017), rice OsALKBH9 (Tang et al., 2024), and tomato SlALKBH2 (Zhou et al., 2019). m^6^A is recognized by m^6^A-binding proteins, such as ECTs in Arabidopsis, which contains the YTH domain. In plants, the ratio of m^6^A/A in poly A^+^ RNA varied among different tissues, with a range of 0.36–0.75% in Arabidopsis and 0.52–0.67% in rice, suggesting its high abundance (Wang et al., 2024). At the transcriptome level, m^6^A sites are primarily enriched within the 3′-untranslated region (3′ UTR), followed by the coding DNA sequence (CDS) and 5′-untranslated region (5′ UTR). Recent studies have demonstrated the crucial roles of m^6^A in regulating gene expression in plants, primarily by influencing mRNA stability, translation, and 3′ UTR processing (Tang et al., 2023). Among them, mRNA stability regulation is one of the primary functions of m^6^A, which involves two aspects: acceleration of RNA decay or preservation of RNA stability, depending on the specific m^6^A-binding proteins. Recent studies combining proteomics and m^6^A analysis have shown that m^6^A in the untranslated regions is negatively correlated with protein abundance, suggesting that m^6^A in UTR is likely to inhibit protein abundance in plants (Li et al., 2024). Therefore, manipulating m^6^A could lead to an increase in protein abundance. m^6^A modifications have also been found to play crucial roles in plant biology, such as embryo development, floral transition, stem cell fate determination, pollen development, fruit ripening, photomorphogenesis, circadian clock, nitrate signaling, and responses to biotic and abiotic stress (Tang et al., 2023). Given the high abundance and crucial roles of m^6^A modifications in gene expression regulating, altering m^6^A modifications in genes holds promise as a strategy for enhancing crop agronomic traits.

## Manipulating m^6^A modification to improve crop agronomic traits

In mammals, the m^6^A demethylase FTO, known as an obesity gene, plays a crucial role in regulating body weight. Researchers have genetically engineered rice and potato to express FTO. In field experiments, the yield and biomass of genetically engineered rice and potato increased by approximately 50% (Yu et al., 2021). Further research indicates that the expression of FTO in rice promotes root growth, tiller bud formation, photosynthetic efficiency, and drought resistance, and these phenotypes are dependent on the m^6^A demethylase activity of FTO (Yu et al., 2021). In strawberries, inhibiting the expression of m^6^A methyltransferase genes *FveMTA* or *FveMTB* can delay fruit ripening, while upregulating the expression of either gene can accelerate fruit ripening, suggesting that manipulating m^6^A modification can regulate the ripening time of strawberries (Zhou et al., 2021). The above research suggests that manipulating the m^6^A modification level overall can enhance the agronomic traits of crops.

In addition to altering the overall m^6^A modification level, researchers have also endeavored to edit m^6^A modification levels on individual genes. In plants, a precise editing system for plant mRNA m^6^A has been successfully developed by fusing dCas13a with the plant m^6^A methyltransferase core complex MTA-MTB or the mammalian demethylase ALKBH5 (Shi et al., 2024). By specifically editing the m^6^A modification on the *SHR* transcript, which is a key gene for root development, it was found that an increase in the m^6^A modification level of the *SHR* transcript can promote the enlargement of the aboveground and root parts of plants, increase leaf area, plant height, biomass, and grain yield, thereby promoting plant growth (Shi et al., 2024). In cotton, similar m^6^A editing tools have also been developed, combining CRISPR/dCas13(Rx) with the methyltransferase GhMTA (Targeted RNA Methylation Editor, TME) or the demethyltransferase GhALKBH10 (Targeted RNA Demethylation Editor, TDE) (Yu et al., 2024). Using TME editor, the m^6^A level of *GhDi19* transcript increased, and the plants with increased m^6^A levels results in a significant increase in root length and enhanced drought resistance. Both works indicate that manipulating the m^6^A modification level of key genes can regulate plant phenotype and improve agronomic traits.

## Strategies for improving crop traits through m^6^A modification

According to current research, three strategies have been proposed for enhancing crop agronomic traits through m^6^A modification. (1) Altering RNA m^6^A modification regulatory proteins (RMRPs) (methyltransferases, demethylases, and recognition proteins) and their interacting proteins. Given that m^6^A regulatory proteins typically regulate a multitude of substrate genes, this strategy exhibits a high degree of randomness and uncertainty in improving agronomic traits, and may frequently result in other phenotypic abnormalities; (2) Conducting m^6^A editing by fusing Cas13 (dCas13) with RNA-modified methyltransferase or demethylase in specific mRNA. However, the RNA-based m^6^A editing strategy poses limitations in crop breeding applications: the m^6^A editing vector must be maintained in the offspring, otherwise the editing effect of m^6^A cannot be preserved. Currently, they are not suitable for practical breeding applications; (3) Editing m^6^A motif of specific gene to remove m^6^A modifications. For example, using a base editor to accurately replace m^6^A-modified adenine on DNA. Regarding the m^6^A modification site on the UTR, the CRISPR/Cas9 system can be employed to disrupt the m^6^A modification motif on the UTR, thereby eliminating the m^6^A modification. This strategy involves directly altering m^6^A motifs on DNA, enabling the production of m^6^A editing materials without transgenic vectors in offspring, which can be utilized in breeding applications.

## Steps for m^6^A editing

### Steps 1: drawing single-base resolution m^6^A modification map

**Figure 1:**
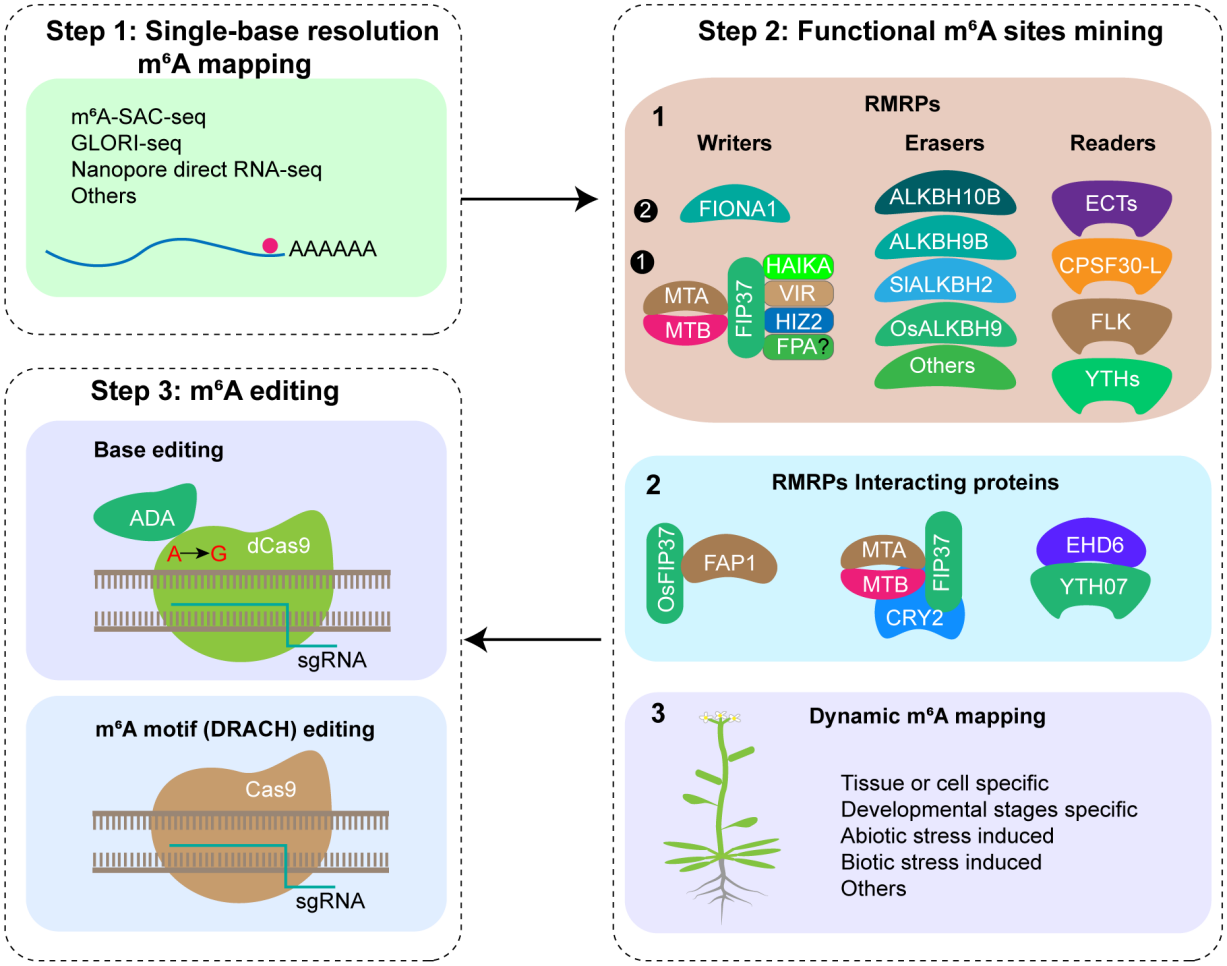
Putative steps of m^6^A editing in crop traits improvement. Step 1: Mapping mRNA m^6^A modification at single-base resolution; Step 2: Functional m^6^A sites mining by functional study of RMRPs, RMRPs interacting proteins, and dynamic m^6^A modification mapping; Step 3: m^6^A editing using CRISPR/Cas9 system or base editor.

For the purpose of m^6^A editing, accurate m^6^A modification maps at single-base resolution must be generated initially. With the advancements in m^6^A detection technology, various single base resolution m^6^A sequencing methods have been reported, among them, m^6^A-selective allyl chemical labeling and sequencing (m^6^A-SAC-seq) (Hu et al., 2022), Nanopore direct RNA sequencing (DRS) (Zheng et al., 2020) and Glyoxal and nitrite-mediated deamination of unmethylated adenosine and sequencing (GLORI-seq) (Liu et al., 2022) are three methods that have broad application prospects (**Figure 1**). Recent research has reported the transcriptome-wide m^6^A maps at single-base resolution in various tissues of rice (plumule dark, plumule light, seedling at 8 days, seedling at 2 weeks, panicle, flag leaf at 10 days after anthesis, endosperm at 10 days after anthesis, and embryo at 10 days after anthesis) and Arabidopsis (seedling, shoot, root, rosette leaf, cauline leaf, stem, flower, silique, and seed) using m^6^A-SAC-seq, identifying a total of 205,691 m^6^A sites distributed across 22,574 genes in rice, and 188,282 m^6^A sites across 19,984 genes in Arabidopsis, offering comprehensive resources for investigating single-base resolution m^6^A sites and their functions in plants (Wang et al., 2024). Given the advancements in SAC-seq, DRS, and GLORI-seq, there are no technical limitations to m^6^A single base resolution sequencing, and single-base resolution maps for other crops can be constructed rapidly.

### Step 2: identification of functional m^6^A sites

Since there are more than ten thousands m^6^A sites present in the plant transcriptome, which ones have regulatory functions remains a key question to be clarified. Currently, three strategies are employed for identifying functional m^6^A sites (**Figure 1**). (1) Functional study of RMRPs. By utilizing mutants, RNA interference, or overexpression techniques for RMRPs, analyzing the phenotypes of relevant plant materials at different growth and development stages and in response to various biotic and abiotic stresses. Next, m^6^A sequencing and corresponding molecular biology methods are employed to ultimately confirm the m^6^A modification sites associated with the observed phenotypes. Utilizing RMRPs, several m^6^A modification sites with crucial functions have been identified, such as m^6^A in *STM* and *WUS* mRNA regulating shoot stem cell fate in Arabidopsis (Shen et al., 2016), *FT*, *SPL3*, and *SPL9* mRNA regulating floral transition in Arabidopsis (Duan et al., 2017), and *OsYUCCA3*, *TDR*, and *GAMYB* mRNA regulating rice pollen development (Cheng et al., 2022; Tang et al., 2024), etc. In the future, we should enhance the application of genetic materials, comprehensively study their functions under various growth and development stages, environmental stress and stimulation, and different pest and disease stress in the field, and explore important functional m^6^A sites. (2) Functional study of proteins interacting with RMRPs. Interaction proteins of RMRPs might be involved in the processes of installing, removing, or recognizing m^6^A modifications at specific sites on specific mRNAs. Several studies have demonstrated the selection of specific m^6^A sites by interacting proteins of RMRPs. In Arabidopsis, CRY2 recruits the m^6^A writer complex (MTA/MTB/FIP37) to the photobody under blue light, thereby promoting m^6^A modification of circadian clock associated 1 (*CCA1*) and enhancing its mRNA stability (Wang et al., 2021). In rice, OsFIP37 is recruited by the RNA-binding protein OsFAP1 to deposit the m^6^A modification on *OsYUCCA3* mRNA, thereby regulating male germ cell miosis (Cheng et al., 2022). EARLY HEADING DATE6 (EHD6) recruits the m^6^A reader YTH07 and sequesters *OsCOL4* mRNA into phase-separated ribonucleoprotein condensates, thereby promoting rice flowering (Cui et al., 2024). (3) Exploring key m^6^A modification sites through dynamic m^6^A modification mapping. By mapping the changes in m^6^A modification maps under various tissues, developmental stages of the same tissue, and stress treatments of crops, m^6^A modification sites specific to corresponding processes are expected to be identified, which may have relevant regulatory functions.

### Step 3: m^6^A editing using CRISPR/Cas9 system or base editor

After generating a single-base resolution m^6^A modification map and identifying functional m^6^A modification sites, m^6^A motif of specific gene can be edited using the CRISPR/Cas9 system or base editor (**Figure 1**). After gene editing, screening transgenic positive seedlings, identifying mutations, and confirming m^6^A modification changes, m^6^A-edited plants were obtained. Subsequently, phenotype analysis will be conducted and transgenic free plants will be screened for subsequent breeding applications.

## Discussion

Fine-tuning gene expression is of great significance for crop improvement. m^6^A, the most abundant modification in mRNAs, plays crucial roles in regulating gene expression, positioning it as a potential tool for manipulating gene expression. With the advent of new m^6^A sequencing methods, mapping m^6^A at single base resolution is no longer an obstacle. The primary limitation of m^6^A application is the insufficient mining of functional m^6^A sites. The most effective approach to explore functional m^6^A sites in crops involves studying the biological functions of m^6^A methyltransferases, demethylases, or m^6^A binding proteins in crop development, biotic and abiotic stress response, and other processes. However, fewer RMRPs have been identified in crops so far, and future research should strengthen the identification and functional research of crop RMRPs. In summary, because of the vital roles of m^6^A in regulating gene expression post-transcriptionally, the m^6^A editing strategy holds significant potential for crop improvement.
